# Long-term Success and Follow-up After Atrial Fibrillation Ablation

**DOI:** 10.2174/157340312803760758

**Published:** 2012-11

**Authors:** S Kircher, G Hindricks, P Sommer

**Affiliations:** University of Leipzig. Heart Center, Department of Electrophysiology, Struempellstr. 39, 04289 Leipzig, Germany

**Keywords:** Atrial fibrillation, catheter ablation, success rate, asymptomatic recurrences, follow-up strategies, implantable cardiac monitors.

## Abstract

Atrial fibrillation (AF) is the most prevalent sustained arrhythmia in clinical practice. It is associated with significant morbidity and mortality and has been identified as an independent risk factor for ischemic stroke and thromboembolic events. Catheter ablation has become an established rhythm control therapy in patients with highly symptomatic drug-refractory AF. The definition of ablation success remains controversial since current symptom-based or intermittent electrocardiogram monitoring strategies fail to sufficiently disclose rhythm outcome. This failure is mainly related to the high incidence of asymptomatic AF recurrences, the unpredictable nature of arrhythmia relapses, and the poor correlation of symptoms and AF episodes. There is a clear correlation between the intensity of the monitoring strategy and the sensitivity for it to detect arrhythmia recurrences. Furthermore, several clinical studies assessing the long-term efficacy of catheter ablation procedures have reported late AF recurrences in patients who were initially considered responders to catheter ablation. In certain subsets of patients, precise long-term monitoring may help to guide therapy, e.g. patients in whom withdrawal of antithrombotic therapy may be considered if they are free of arrhythmia recurrences. Recently, sub-cutaneous implantable cardiac monitors (ICM) have been introduced for prolonged and continuous rhythm monitoring. The performance of a leadless ICM equipped with a dedicated AF detection algorithm has recently been assessed in a clinical trial demonstrating a high sensitivity and overall accuracy for identifying patients with AF. The clinical impact of ICM-based follow-up strategies, however, has to be evaluated in prospective clinical trials.

## INTRODUCTION

Atrial fibrillation (AF) is the most common sustained cardiac rhythm disturbance in clinical practice affecting 1-2 % of the general population with the prevalence increasing with age [1,2]. The number of patients with AF is estimated to increase approximately 2.5 fold within the next 50 years [3]. AF is a major cardiac cause of morbidity that is primarily related to a reduced quality of life, heart failure and thromboembolic events, and thus represents a considerable health care burden [4-7]. During the past 20 years, hospital admissions for AF have substantially increased which is mainly attributed to the ageing of the population, a higher prevalence of chronic heart disease, and more frequent diagnosis through use of arrhythmia monitoring devices [8]. According to data from the Framingham Heart Study, AF is independently associated with an increased mortality [9]. Management of AF encompasses antithrombotic treatment according to the individual stroke risk and either rate control or rhythm control strategies [1,8,10]. The decision to accept AF and control ventricular response or to restore and maintain sinus rhythm is mainly driven by the presence and severity of symptoms, the type of AF, patients´ preference, and a variety of clinical factors such as age, cardiovascular co-morbidities and left atrial size. During the last decade, catheter ablation of AF has evolved from a highly experimental procedure to a standard rhythm control strategy for patients with symptomatic AF [1,11]. There is general agreement that pulmonary vein isolation forms the cornerstone of any ablation procedure. Whilst standardized procedural endpoints have been established, the definition of “long-term ablation success” remains controversial. This is primarily related to the inability of current post-interventional rhythm monitoring strategies to provide valid detection of AF recurrences or secondary arrhythmias (i.e. atrial tachycardia and atypical atrial flutter). This review focuses on the clinically relevant aspects of current follow-up strategies after catheter ablation for AF and their implications on “ablation success”.

## RHYTHM MONITORING AFTER CATHETER ABLATION: GENERAL CONSIDERATIONS

From a pragmatic point of view one might argue that a follow-up strategy exclusively based on symptom reporting is sufficient to define ablation success since (1) catheter ablation primarily aims at the elimination of symptoms and the improvement of quality of life and (2) long-term anticoagulation should be managed according to the individual stroke risk [1,11,12]. However, a more objective follow-up strategy based on regular scheduled or continuous ECG monitoring may be required in both clinical care and clinical research trials for several reasons. First, the diagnostic yield of symptom-based follow-up strategies is substantially limited not only because of the reported high incidence of silent arrhythmia recurrence (see below), but also due to the well-known poor symptom-arrhythmia correlation and the unpredictability of AF recurrences [13-18]. In a considerable number of patients, palpitations resulting from atrial or ventricular premature complexes, or sinus tachycardia may be misinterpreted as AF episodes. In a study by Israel *et* *al*., 40 % of patients with an implanted pacemaker reported symptoms suggestive of AF but standard ECG recording and device interrogation proved absence of AF during the respective episode [15]. In a study by Quirino *et al*. that included 89 patients with a history of paroxysmal AF and permanent pacemaker implantation for sick sinus syndrome, only 240 (21 %) out of 1,141 patient reported episodes corresponded to a device-stored AF recurrence [13]. In a subgroup analysis of the Mode Selection Trial (MOST) that included 312 patients with permanent pacemakers, the sensitivity of symptom reporting was 82.4 %, whereas specifity measured 38.3 % with a positive predictive value of only 58.7 % [16]. Second, a reliable assessment of rhythm outcome is essential for the comparison of different rhythm control strategies and the definition of success in both clinical care and research trials [19]. Appropriate surveillance during the early post-ablation period may identify patients who are at higher risk of long-term treatment failure since early recurrences strongly predict a lack of long-term success [20,21]. Third, early detection of silent AF relapses and / or secondary arrhythmias may be of great importance to prevent the development of a tachycardia-induced cardiomyopathy resulting from unrecognized high ventricular rates [22]. Finally, valid rhythm monitoring may help to guide clinical decision making in certain subsets of patients. In a study by Botto *et al*. that included 568 patients with permanent pacemakers and a history of AF, the incidence of thromboembolic events was 0.8 % per year in patients with a CHADS_2_-score ≤ 2 and AF recurrences with a maximum duration of 5 minutes on device interrogation [23]. This data suggests that a highly accurate follow-up, as provided by implantable cardiac devices, may add valuable information to current clinical risk stratification schemes. Thus, it might be reasonable to discontinue oral anticoagulation in selected patients with a low to moderate risk for thromboembolic events and freedom from AF or atrial tachycardia as evidenced by a rhythm monitoring strategy allowing for reliable conclusions about rhythm control.

## EPIDEMIOLOGY OF ASYMPTOMATIC ATRIAL FIBRILLATION

The clinical presentation of AF is highly variable and frequently difficult to predict in an individual patient. The arrhythmia is typically associated with a variety of symptoms including palpitations, limited exercise capacity, chest pain, dyspnea, fatigue, dizziness and syncope [1,6]. However, AF may recur without clinical signs or symptoms in a significant proportion of patients. Older age, persistent or permanent AF, male sex and lower ventricular rates have been associated with an increased risk of silent episodes, but data from different studies are inconsistent implying that clinical factors and arrhythmia pattern cannot reliably predict patients with silent AF episodes [1,6,24-29]. Detection of silent AF episodes in individual patients is even more troublesome as symptomatic and asymptomatic episodes may coexist in the same patient [30,31]. Page *et al*. reported that silent AF episodes were detected in 17 % of patients before the recurrence of any symptomatic supraventricular arrhythmia in patients with a history of symptomatic AF or atrial flutter [31]. Furthermore, medical rhythm control or rate control may convert symptomatic AF into silent AF by altering arrhythmia perception [32,33]. There is great evidence that the burden of AF may be grossly underestimated due to the high incidence of subclinical AF episodes [34,35]. It is appreciated that silent episodes occur in at least one third of AF patients [6]. The reported incidence of asymptomatic AF strongly depends upon the intensity of rhythm monitoring, the duration of the follow-up period, the definition of AF and the AF burden in the respective patient population [34,35]. In studies including elderly patients using “low intensity” methods of rhythm monitoring based on 12-lead ECG, self-reporting, physical examination, hospital discharge diagnoses and single 24-hour Holter monitoring, the reported incidence of silent AF varied between 10 and 40 % [29,36-40]. In contrast, extended rhythm monitoring with a cardiac event recorder allowing for continuous automatic rhythm analysis and storage revealed that 55 % of patients with documented paroxysmal atrial fibrillation and a negative 24-hour Holter monitoring were asymptomatic [41]. Implantable cardiac devices, such as permanent pacemakers and defibrillators with dedicated atrial arrhythmia detection algorithms, allow for continuous rhythm monitoring with a high sensitivity and specificity [15,42,43]. In the Automatic Interpretation for Diagnosis Assistance (AIDA) study, 58 % of patients with a permanent pacemaker for sinus node dysfunction and / or atrioventricular node disease and a history of clinical AF had silent AF episodes lasting longer than 1 minute detected by the device during a 1-month follow-up period [42]. Israel *et al*. assessed the incidence of AF recurrences in 110 patients with permanent pacemakers and a history of paroxysmal or persistent AF [15]. After a mean observation period of 19 ± 11 months, AF was detected in 51 patients (46 %) by resting ECG recording and in 97 patients (88 %) by device interrogation. AF episodes lasting longer than 48 hours were found in 50 patients, 19 of whom (38 %) were asymptomatic.

## CLINICAL IMPLICATIONS OF ASYMPTOMATIC ATRIAL FIBRILLATION

AF is an independent risk factor for ischemic stroke [44]. Strokes related to AF confer a higher risk of death and permanent disability than strokes of other causes [45,46]. The use of warfarin significantly reduces the risk of stroke as demonstrated in a meta-analysis of several randomized trials [47]. There is growing evidence that silent AF is associated with a relevant risk of thromboembolic events [16,48,49,50]. In the Atrial Fibrillation Follow-up Investigation of Rhythm Management (AFFIRM) study, 5.0 % of patients in the rate control group and 7.1 % in the rhythm control group experienced an ischemic stroke [48]. In 55 % of patients in the rhythm control arm and in 36 % of patients under rate control, the ischemic event occurred after discontinuation of warfarin. The higher stroke rate in the rhythm control group may at least partially be explained by the presence of silent AF episodes in patients in whom AF was considered to be suppressed and thus anticoagulation was withdrawn. A sub-analysis of the AFFIRM study demonstrated that silent AF did not confer a better outcome with respect to mortality and major events after adjustment for baseline characteristics [49]. A subgroup analysis of MOST that included 312 patients who had permanent pacemaker implantation for sinus node dysfunction, was designed to assess the clinical significance of atrial high rate episodes (AHRE) as detected by pacemaker diagnostics [16]. Pacemakers were programmed to store an AHRE when the atrial rate was > 220 beats per minute. Analyses were confined to patients with AHRE lasting longer than 5 minutes. After a median follow-up of 27 months, 160 out of 312 patients (51.3 %) had at least one AHRE. The presence of any AHRE was an independent predictor of total mortality, death or non-fatal stroke, and AF. More importantly, almost one fifth of patients with AHRE were asymptomatic. The recently published Asymptomatic Atrial Fibrillation and Stroke Evaluation in Pacemaker Patients and the Atrial Fibrillation Reduction Atrial Pacing Trial (ASSERT) prospectively evaluated the association of subclinical atrial high-rate episodes detected by implanted devices (i.e. pacemakers and defibrillators) and the risk of ischemic stroke in 2580 patients aged ≥ 65 years with a history of hypertension and no prior diagnosis of clinical AF [50]. Patients were initially monitored for 3 months to detect subclinical AHRE which were defined as episodes of atrial rate > 190 beats per minute lasting more than 6 minutes. Patients were subsequently followed for a mean of 2.5 years for the primary outcomes of ischemic stroke or systemic embolism. After 3 months, subclinical atrial tachyarrhythmias were recorded in 261 patients (10.1 %). Subclinical atrial tachyarrhythmias were independently associated with a 2.5-fold increase in the risk of subsequent ischemic stroke or systemic embolism.

## ASYMPTOMATIC AF AFTER CATHETER ABLATION

Patients who are considered for AF catheter ablation usually represent a highly symptomatic subgroup within the general AF population. Several studies, however, demonstrated the occurrence of asymptomatic AF episodes during the follow-up period after ablation in a substantial number of patients [51-54]. Vasamreddy *et al.* monitored 19 consecutive patients after catheter ablation with mobile cardiac outpatient telemetry (MCOT) that allowed for automatic arrhythmia detection and ECG transmission to a service center [52]. During the follow-up period, MCOT was applied for 5 days per month, for 6 consecutive months after the ablation. Additionally, patients were asked to activate the system when symptoms they attributed to AF occurred. After 6 months, 7 out of 10 patients (70 %) with a complete follow-up were free from symptomatic AF recurrences. However, the success rate decreased to 50 % when silent AF episodes were considered. In 80 consecutive patients with paroxysmal AF studied by Klemm *et al.,* post-procedural follow-up consisted of daily and symptom-activated transtelephonic ECG transmissions for 6 months [53]. In total, 6,835 transmitted episodes were analyzed. Sinus rhythm was present in 5,437 episodes (79.5 %) whereas AF was recorded in 1,398 episodes (20.5 %) with 752 AF episodes (53.8 %) being clinically silent. Seven out of 80 patients (8.8 %) with AF recurrences were completely asymptomatic. Hindricks *et al.* prospectively evaluated the incidence of asymptomatic AF episodes in 114 patients with highly symptomatic, drug-refractory AF who were selected for catheter ablation [54]. Serial continuous 7-day Holter ECG recordings were performed prior to the procedure, immediately after ablation, and after 3, 6, and 12 months of follow-up respectively. Before ablation, 92 of 114 patients (81 %) had AF episodes. Both symptomatic and asymptomatic AF episodes were observed in 52 patients (57 %), whereas 5 patients (5 %) exclusively experienced silent AF episodes. After the procedure, the percentage of patients with only asymptomatic AF relapses significantly increased to 38 %, 37 %, and 36 % after 3, 6, and 12 months of follow-up, respectively. Analyses of patient characteristics and arrhythmia patterns could not identify specific subsets of patients who were at an increased risk for silent AF. This significant post-interventional increase in the number of patients with asymptomatic AF episodes may be partially explained by changes in arrhythmia perception due to a placebo-effect which has been observed after other invasive procedures [55] and ablation-induced modulation of the autonomic nervous system.

## RHYTHM MONITORING AFTER CATHETER ABLATION

In the recent years, several randomized trials comparing the efficacy of catheter ablation against antiarrhythmic drug treatment have been published reporting freedom from AF after ablation in 57 % to 89 % of patients after one year of follow-up [56-59]. Data from several studies suggest that differences in the reported rhythm outcome after catheter ablation are not only related to patient selection, ablation technique and experience but also due to the different post-interventional rhythm monitoring strategies [60-63]. Furthermore, there is a clear positive correlation between the duration and intensity of the follow-up and the arrhythmia detection rate, suggesting that the reported outcome after catheter ablation is substantially overestimated. Kottkamp *et al.* investigated the diagnostic yield of different rhythm monitoring durations in 100 patients who underwent circumferential pulmonary vein isolation and substrate modification for highly symptomatic paroxysmal and persistent AF [60]. In patients with paroxysmal AF, significantly more AF recurrences were detected by 7-day Holter monitoring immediately after ablation as well as at three and six months follow-up as compared to the conventional approach using 24-hour ECG recording. At 12 months follow-up, there was still a numeric difference between both strategies with 88 % of patients being free from AF relapses on 24-hour ECG Holter as compared to 74 % of patients with 7-day Holter ECG recording. Similar results were reported by Dagres *et al*. who evaluated the diagnostic yield of different Holter durations in 215 consecutive patients with 7-day Holter monitoring 6 months after AF catheter ablation [61]. Compared to the complete 7-day ECG recording period, any Holter duration equal to or lower than 5 days would have detected significantly less patients with AF relapses. The proportion of patients with AF recurrences would have increased from 59 % using 24-hour Holter ECG to 91 % when Holter ECG duration was extended to 96 hours. In another study by Senatore *et al.*, a follow-up strategy consisting of 24-hour Holter ECG and standard ECG recording performed one and four months after ablation was compared against daily and symptom-driven 30-second transtelephonic ECG transmissions starting one month after ablation in 72 patients during a short-term follow-up period of 4 months after catheter ablation [62]. During the follow-up period, transtelephonic ECG monitoring detected a significantly higher number of patients with AF recurrences compared to the approach based on Holter and ECG recording (27.8 % versus 13.9 %). Piorkowski *et al*. prospectively compared the diagnostic yield of serial 7-day Holter recordings against transtelephonic ECG recording in 30 consecutive patients with highly symptomatic AF after catheter ablation [63]. In all patients, a continuous 7-day Holter ECG was performed prior to the procedure, immediately after the procedure, and after 3 and 6 months respectively. Additionally, 12 lead ECGs were routinely transtelephonically transmitted every 2 days throughout the 6-month follow-up period and on occurrence of symptoms suggestive of AF recurrences. Using a follow-up strategy based solely on symptoms, the success rate, defined as freedom from AF, would have been reported as 70 % after a blanking period of one or three months. With serial 7-day Holter recordings and transtelephonic ECG transmission strategies the success rates declined to 50 % and 45 % of patients, respectively.

Several studies reported on the rate of late AF recurrences, defined as AF recurrences 12 months or more after the ablation procedure [64-69]. Weerasooriya *et al*. assessed the long-term efficacy of catheter ablation procedures in 100 patients with paroxysmal or persistent AF using serial 24-hour Holter ECG monitoring and symptom-initiated ECG recording [64]. After a single catheter ablation procedure, complete ablation success, defined as absence of any AF or atrial tachycardia recurrence lasting at least 30 seconds, was achieved in 40 %, 37 %, and 29 % at 1, 2, and 5 years, respectively. After a median of 2 procedures per patient, arrhythmia-free survival following the last ablation procedure was observed in 87 %, 81 %, and 63 % of patients at 1, 2, and 5 years, respectively. In a study by Sorgente *et al*., 103 patients were followed for a median of 6 years to evaluate ablation outcome [65]. Freedom from any atrial tachycardia was present in 23 % of patients after a single procedure and in 39 % after the last procedure. About two thirds of recurrences occurred within 12 months after the ablation procedure. Martinek *et al*. followed 14 patients with implanted pacemakers capable of arrhythmia detection and storage in order to assess various aspects of very long-term outcome (mean follow-up = 41.4 ± 15.1 months) [66]. Successful response to catheter ablation was defined as a decrease of atrial tachyarrhythmia burden to less than 10 minutes per day in the first 24 months. The proportion of responders declined from 71 % when follow-up was solely based on symptom-reporting to 57 % when 7-day Holter recording was applied. Pacemaker diagnostics revealed a responder rate of 43 %, with only 21 % of patients being free from any tachyarrhythmia episode. Bertaglia *et* *al*. followed 177 patients who were considered free from any atrial tachyarrhythmia recurrence during the first year after ablation (67). During a mean follow-up period of 49.7 ± 13.3 months, AF recurrences were detected in 74 out of these 177 patients (41.8 %).

## CURRENT STANDARDS AND FUTURE DIRECTIONS

**Fig. (1):**
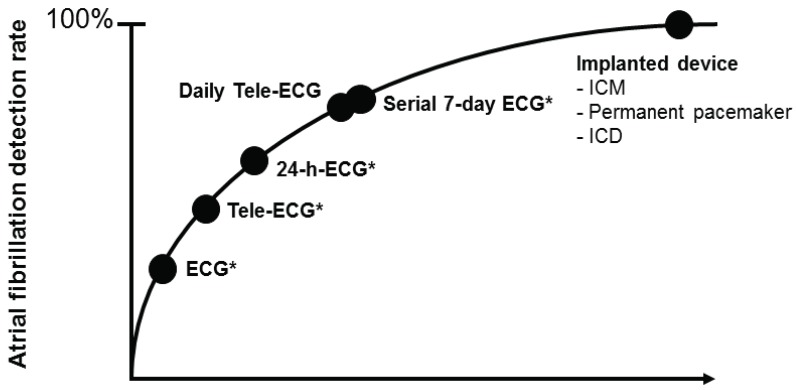
Estimated correlation between the intensity of the follow-up strategy and atrial fibrillation detection rate (Modified from [75]). *During quarterly follow-up visits; ICM: Implantable cardiac monitor; ICD: Implantable cardioverter-defibrillator

**Fig. (2):**
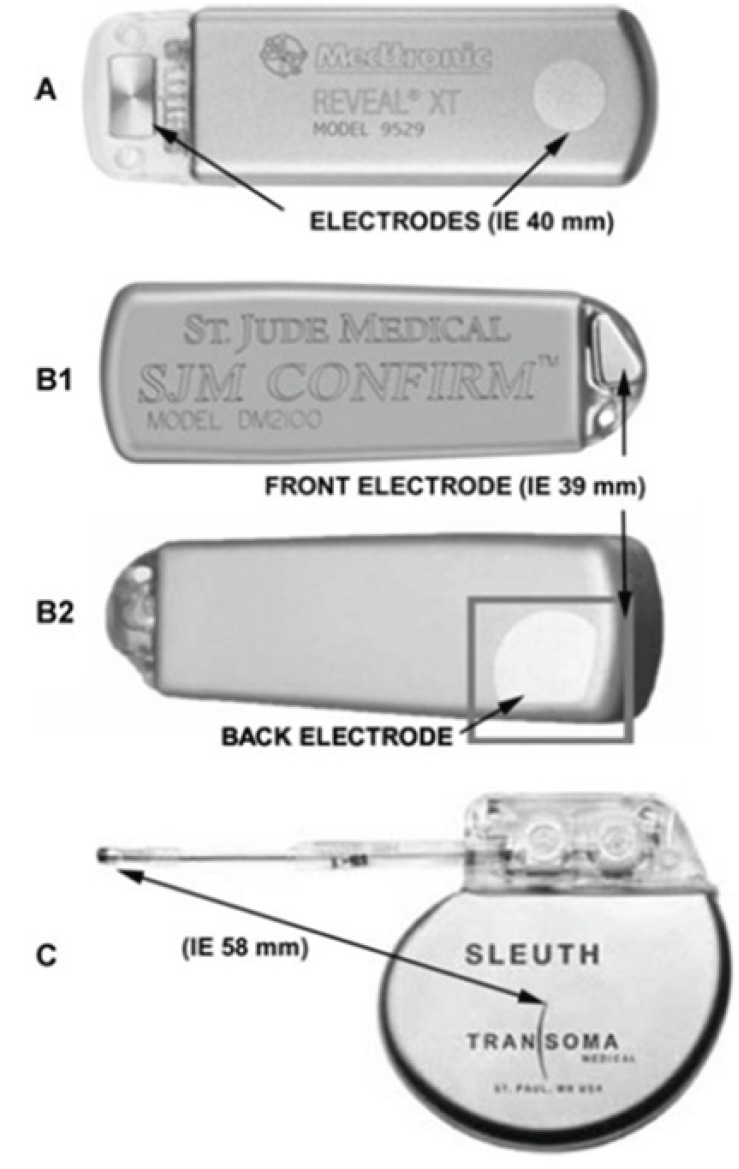
Available implantable cardiac monitors. (**A**) Reveal® XT (Medtronic), (**B1**) and (**B2**) Confirm® (St. Jude Medical), and (**C**) Sleuth™ (Transoma), equipped with integrated (**A** and **B**) or external (**C**) electrodes.

Expert consensus recommends a follow-up strategy consisting of standard ECGs obtained at scheduled follow-up visits for at least two years after catheter ablation [11]. A more intense follow-up should be driven mainly by the clinical impact of AF detection. Currently, follow-up strategies are mainly based on non-continuous rhythm monitoring tools including scheduled or symptom-triggered standard ECGs, Holter ECG monitoring (24 hours to 7 days), patient and automatically activated event recorders, external loop recorders and transtelephonic ECG transmissions. Any of these strategies bears device specific limitations including unsatisfactory description of arrhythmia episodes in the case of short-term recordings and poor patient compliance in the case of more intense monitoring [52]. The most important limitations of intermittent monitoring, however, are the deficient sensitivity and negative predictive values (Fig. **1**). Ziegler *et al.* retrospectively investigated the diagnostic yield of symptom-based and intermittent monitoring compared to continuous rhythm monitoring provided in 574 patients with permanent pacemakers over a follow-up period of one year [35]. Non-continuous monitoring i.e. annual, quarterly, and monthly 24-hour Holter; 7-day and 30-day annual long-term recordings, was simulated by analyzing data from randomly selected days within a pre-defined monitoring window. Symptom-based monitoring was approximated by analyzing days when patients reported symptoms by means of an external activator. Compared with continuous monitoring, symptom-based and all intermittent monitoring strategies were associated with a significantly lower sensitivity (range 31 %-71 %) and negative predictive value (range 21 %-39 %) for detection of patients with any atrial tachyarrhythmia. In a study by Botto *et al.* that included 568 patients with a permanent pacemaker and prior AF episodes, the mean sensitivity in detecting AF episodes lasting longer than 5 minutes measured 44.4 %, 50.5 %, and 65.1 % for 24-hour Holter ECG, 1-week Holter ECG, and 1-month Holter ECG monitoring, respectively [23]. Highly reliable rhythm surveillance would be desirable in certain patient subgroups to guide therapy, e.g. in patients at low to moderate risk of thromboembolic events in whom discontinuation of antithrombotic therapy may be considered if they are arrhythmia free after ablation. Continuous rhythm monitoring, as provided by implantable pacemakers and defibrillators with an atrial lead and the capability of arrhythmia detection, storage, and quantification, has been shown to be highly sensitive and specific. However, these tools are limited to a small group of AF patients with a standard indication for device therapy [43,70]. In the recent years, subcutaneous implantable cardiac monitors (ICM) have been developed for continuous rhythm assessment (Fig. **2**). ICMs have been incorporated into the current guidelines as diagnostic tools for the evaluation of patients with unexplained syncope [71]. The Reveal XT Performance Trial (XPECT) prospectively assessed the short-term performance of a leadless ICMs equipped with a dedicated AF detection algorithm in 247 patients with paroxysmal AF [72]. The AF detection algorithm, which was designed to detect the presence of AF and AF burden, uses the irregularity and incoherence of R-R intervals to identify and classify patterns of ventricular conduction. The R-R intervals are assessed within each 2-minute period of time, and the variability of the differences in R-R intervals is calculated. Episodes are classified as AF when the R-R intervals within the 2-minute interval show a certain pattern of uncorrelated irregularity. The ICM is capable of storing up to 49.5 minutes of recorded ECGs, which are allocated to 22.5 minutes of patient-activated events and 27 minutes of automatically stored episodes. Additionally, the ICM has an episode log that allows for storing of 30 automatically detected episodes and up to 10 patient-initiated events. When storage capacity is exhausted, an additional episode will overwrite the oldest stored episode. The ICM automatic arrhythmia classification was compared to the analysis of a simultaneously recorded 46-hour Holter ECG. Complete data were available for 206 patients and demonstrated a sensitivity, specificity, positive predictive value, and negative predictive value of 96.1 %, 85.4 %, 79.3 %, and 97.4 %, respectively, for identifying patients with any AF episode. The AF burden was accurately measured by the ICM in the vast majority of patients. False-positive classification by the ICM was related to frequent premature atrial or ventricular complexes, oversensing due to myopotentials, irregular sinus rhythm, bigeminy and other atrial arrhythmias. The superiority of continuous rhythm monitoring provided by an ICM over intermittent recording strategies has been demonstrated in 45 patients after surgical ablation who were followed by an ICM and quarterly 24-hour Holter ECG recording [73]. In patients with a low AF burden, 24-hour Holter ECG monitoring substantially underestimated the “true” incidence of AF recurrences as evidenced by the ICM. In a study by Eitel *et al.*, there was a trend towards a higher AF detection rate on ICM compared against serial 7-day Holter monitoring in 51 patients after catheter ablation [74]. In another study by Pokushalov *et al.*, ICM recording was used to guide postinterventional management in 268 patients who underwent catheter ablation for paroxysmal AF [75]. Patients with AF recurrences within the first 3 months after ablation were randomly assigned into two treatment groups. Patients in group 1 received antiarrhythmic treatment for 6 weeks and re-ablation was performed only in the event of AF recurrences after the 3-months interval. In group 2, patients were treated according to the mode of AF initiation as detected and stored by the ICM [76]. If AF recurrences were preceded by triggers (e.g. premature atrial complexes, atrial tachycardia), patients were scheduled for a repeat ablation procedure. If no triggers were identified, an antiarrhythmic therapy was initiated. At 12-month follow-up, significantly more patients in group 1 had AF recurrences as compared to group 2 (67 % versus 20 %).

Follow-up strategies based on the currently available ICMs have major shortcomings because of the limited electrogram storage capacity, the high burden of non-diagnostic interrogations and the exclusion of short-lasting AF. These limitations may be overcome by individual device programming and remote device interrogation aiming at reducing storage overflow.

## CONCLUSION

Catheter ablation for AF has become a standard rhythm control therapy in patients with highly symptomatic drug-refractory AF. The definition of ablation success remains controversial since currently applied rhythm detection strategies are based on symptom-reporting and intermittent ECG recording and thus substantially underestimate the “true” rate of AF recurrences. Data from clinical trials suggest the existence of AF recurrences even after an “arrhythmia-free” interval of 12 months after the ablation procedure. Therefore, in certain subsets of patients, a precise and sufficiently long rhythm monitoring may be required to adequately guide individual clinical decision making. Recently, a subcutaneous leadless ICM has been validated in a clinical trial demonstrating a high sensitivity and overall accuracy for detecting AF. Continuous rhythm monitoring based on ICM may allow for the development of new follow-up standards for both clinical and scientific purposes. Furthermore, most of the data concerning rhythm monitoring after catheter ablation were derived from studies that had been initiated several years ago. Since there have been significant technological improvements, these data may underestimate the efficacy of current ablation approaches. Further prospective studies are required to evaluate the potential impact of ICM based monitoring on clinical care and patient treatment, and on the “true” outcome of current ablation strategies.

## ABBREVIATIONS

AF: = Atrial fibrillation
AHRE: = Atrial high rate episodes
ECG: = Electrocardiogram
ICM: = Implantable cardiac monitor
MCOT: = mobile cardiac outpatient telemetry

## References

[1] Camm AJ, Kirchhof P, Lip GY (2010). European Heart Rhythm Association, European Association for Cardio-Thoracic Surgery, Guidelines for the management of atrial fibrillation: the Task Force for the Management of Atrial Fibrillation of the European Society of Cardiology (ESC). Europace.

[2] Stewart S, Hart CL, Hole DJ, McMurray JJ (2001). Population prevalence, incidence, and predictors of atrial fibrillation in the Renfrew/Paisley study. Heart.

[3] Go AS, Hylek EM, Phillips KA (2001). Prevalence of diagnosed atrial fibrillation in adults: national implications for rhythm management and stroke prevention: the AnTicoagulation and Risk Factors in Atrial Fibrillation (ATRIA) Study. JAMA.

[4] Thrall G, Lane D, Carroll D, Lip GY (2006). Quality of life in patients with atrial fibrillation: a systematic review. Am J Med.

[5] Kirchhof P, Bax J, Blomstrom-Lundquist C (2009). Early and com-prehensive management of atrial fibrillation: proceedings from the 2nd AFNET/EHRA consensus conference on atrial fibrillation enti-tled 'research perspectives in atrial fibrillation'. Europace.

[6] Savelieva I, Camm AJ (2000). Clinical relevance of silent atrial fibrillation: prevalence, prognosis, quality of life, and management. J Interv Card Electrophysiol.

[7] Le Heuzey JY, Paziaud O, Piot O (2004). Cost of care distribution in atrial fibrillation patients: the COCAF study. Am Heart J.

[8] Fuster V, Rydén LE, Cannom DS (2011). 2011 ACCF/AHA/HRS focused updates incorporated into the ACC/AHA/ESC 2006 Guidelines for the management of patients with atrial fibrillation: a report of the American College of Cardiology Foundation/American Heart Association Task Force on Practice Guidelines developed in partnership with the European Society of Cardiology and in collaboration with the European Heart Rhythm Association and the Heart Rhythm Society. J Am Coll Cardiol.

[9] Benjamin EJ, Wolf PA, D'Agostino RB, Silbershatz H, Kannel WB, Levy D (1998). Impact of atrial fibrillation on the risk of death: the Framingham Heart Study. Circulation.

[10] Gage BF, Waterman AD, Shannon W, Boechler M, Rich MW, Radford MJ (2001). Validation of clinical classification schemes for predicting stroke: results from the National Registry of Atrial Fibrillation. JAMA.

[11] Calkins H, Kuck KH, Cappato R (2012). 2012 HRS/EHRA/ECAS Expert Consensus Statement on Catheter and Surgical Ablation of Atrial Fibrillation: Recommendations for Patient Selection, Procedural Techniques, Patient Management and Follow-up, Definitions, Endpoints, and Research Trial Design. Europace.

[12] Oral H, Morady F (2006). How to select patients for atrial fibrillation ablation. Heart Rhythm.

[13] Quirino G, Giammaria M, Corbucci G (2009). Diagnosis of paroxysmal atrial fibrillation in patients with implanted pacemakers: relationship to symptoms and other variables. Pacing Clin Electrophysiol.

[14] Silberbauer J, Veasey RA, Cheek E, Maddekar N, Sulke N (2009). Electrophysiological characteristics associated with symptoms in pacemaker patients with paroxysmal atrial fibrillation. J Interv Card Electrophysiol.

[15] Israel CW, Grönefeld G, Ehrlich JR, Li YG, Hohnloser SH (2004). Long-term risk of recurrent atrial fibrillation as documented by an implantable monitoring device: implications for optimal patient care. J Am Coll Cardiol.

[16] Glotzer TV, Hellkamp AS, Zimmerman J (2003). Atrial high rate episodes detected by pacemaker diagnostics predict death and stroke: report of the Atrial Diagnostics Ancillary Study of the MOde Selection Trial (MOST). Circulation.

[17] Strickberger SA, Ip J, Saksena S, Curry K, Bahnson TD, Ziegler PD (2005). Relationship between atrial tachyarrhythmias and symptoms. Heart Rhythm.

[18] Kirchhof P, Bax J, Blomstrom-Lundquist C (2009). Early and com-prehensive management of atrial fibrillation: proceedings from the 2nd AFNET/EHRA consensus conference on atrial fibrillation enti-tled 'research perspectives in atrial fibrillation'. Europace.

[19] Kirchhof P, Auricchio A, Bax J (2007). Outcome parameters for trials in atrial fibrillation: recommendations from a consensus conference organized by the German Atrial Fibrillation Competence NETwork and the European Heart Rhythm Association. Europace.

[20] Arya A, Hindricks G, Sommer P (2010). Long-term results and the predictors of outcome of catheter ablation of atrial fibrillation using steerable sheath catheter navigation after single procedure in 674 patients. Europace.

[21] Lee SH, Tai CT, Hsieh MH (2004). Predictors of early and late re-currence of atrial fibrillation after catheter ablation of paroxysmal atrial fibrillation. J Interv Card Electrophysiol.

[22] Fenelon G, Wijns W, Andries E, Brugada P (1996). Tachycardiomyopathy: mechanisms and clinical implications. Pacing Clin Electrophysiol.

[23] Botto GL, Padeletti L, Santini M (2009). Presence and duration of atrial fibrillation detected by continuous monitoring: crucial implications for the risk of thromboembolic events. J Cardiovasc Electrophysiol.

[24]  Brand FN, Abbott RD, Kannel WB, Wolf PA (1985). Characteristics and prognosis of lone atrial fibrillation. 30-year follow-up in the Framingham Study. JAMA.

[25] Patten M, Maas R, Karim A, Müller HW, Simonovsky R, Meinertz T (2006). Event-recorder monitoring in the diagnosis of atrial fibrillation in symptomatic patients: subanalysis of the SOPAT trial. J Cardiovasc Electrophysiol.

[26] Patten M, Maas R, Bauer P (2004). Suppression of paroxysmal atrial tachyarrhythmias--results of the SOPAT trial. Eur Heart J.

[27] Boriani G, Padeletti L, Santini M (2007). Rate control in patients with pacemaker affected by brady-tachy form of sick sinus syndrome. Am Heart J.

[28] Lévy S, Maarek M, Coumel P (1999). Characterization of different subsets of atrial fibrillation in general practice in France: the ALFA study. The College of French Cardiologists. Circulation.

[29] Kerr C, Boone J, Connolly S (1996). Follow-up of atrial fibrillation: The initial experience of the Canadian Registry of Atrial Fibrilla-tion. Eur Heart J.

[30] Page RL, Wilkinson WE, Clair WK, McCarthy EA, Pritchett EL (1994). Asymptomatic arrhythmias in patients with symptomatic paroxysmal atrial fibrillation and paroxysmal supraventricular tachycardia. Circulation.

[31] Page RL, Tilsch TW, Connolly SJ (2003). Asymptomatic or "silent" atrial fibrillation: frequency in untreated patients and patients re-ceiving azimilide. Circulation.

[32] Wolk R, Kulakowski P, Karczmarewicz S (1996). The incidence of asymptomatic paroxysmal atrial fibrillation in patients treated with propranolol or propafenone. Int J Cardiol.

[33] Connolly SJ, Schnell DJ, Page RL, Wilkinson WE, Marcello SR, Pritchett EL (2003). Azimilide Supraventricular Arrhythmia Program Investigators. Symptoms at the time of arrhythmia recurrence in patients receiving azimilide for control of atrial fibrillation or flutter: results from randomized trials. Am Heart J.

[34] Rho RW, Page RL (2005). Asymptomatic atrial fibrillation. Prog Cardiovasc Dis.

[35] Ziegler PD, Koehler JL, Mehra R (2006). Comparison of continuous versus intermittent monitoring of atrial arrhythmias. Heart Rhythm.

[36] Camm AJ, Evans KE, Ward DE, Martin A (1980). The rhythm of the heart in active elderly subjects. Am Heart J.

[37] Psaty BM, Manolio TA, Kuller LH (1997). Incidence of and risk factors for atrial fibrillation in older adults. Circulation.

[38] Furberg CD, Psaty BM, Manolio TA, Gardin JM, Smith VE, Rautaharju PM (1994). Prevalence of atrial fibrillation in elderly subjects (the Cardiovascular Health Study). Am J Cardiol.

[39] Kopecky SL, Gersh BJ, McGoon MD (1987). The natural history of lone atrial fibrillation. A population-based study over three decades. N Engl J Med.

[40] Benjamin EJ, Levy D, Vaziri SM, D'Agostino RB, Belanger AJ, Wolf PA (1994). Independent risk factors for atrial fibrillation in a population-based cohort. The Framingham Heart Study. JAMA.

[41] Roche F, Gaspoz JM, Da Costa A (2002). Frequent and prolonged asymptomatic episodes of paroxysmal atrial fibrillation revealed by automatic long-term event recorders in patients with a negative 24-hour Holter. Pacing Clin Electrophysiol.

[42] Defaye P, Dournaux F, Mouton E (1998). Prevalence of supraventricular arrhythmias from the automated analysis of data stored in the DDD pacemakers of 617 patients: the AIDA study. The AIDA Multicenter Study Group. Automatic Interpretation for Diagnosis Assistance. Pacing Clin Electrophysiol.

[43] Swerdlow CD, Schsls W, Dijkman B (2000). Detection of atrial fibrillation and flutter by a dual-chamber implantable cardioverter-defibrillator. For the Worldwide Jewel AF Investigators. Circulation.

[44] Wolf PA, Abbott RD, Kannel WB (1991). Atrial fibrillation as an independent risk factor for stroke: the Framingham Study. Stroke.

[45] Lin HJ, Wolf PA, Kelly-Hayes M (1996). Stroke severity in atrial fibrillation. The Framingham Study. Stroke.

[46] Marini C, De Santis F, Sacco S (2005). Contribution of atrial fibrillation to incidence and outcome of ischemic stroke: results from a population-based study. Stroke.

[47] Hart RG, Pearce LA, Aguilar MI (2007). Meta-analysis: antithrombotic therapy to prevent stroke in patients who have nonvalvular atrial fibrillation. Ann Intern Med.

[48] Wyse DG, Waldo AL, DiMarco JP (2002). A comparison of rate control and rhythm control in patients with atrial fibrillation. N Engl J Med.

[49] Flaker GC, Belew K, Beckman K (2005). Asymptomatic atrial fibril-lation: demographic features and prognostic information from the Atrial Fibrillation Follow-up Investigation of Rhythm Management (AFFIRM) study. Am Heart J.

[50] Healey JS, Connolly SJ, Gold MR (2012). Subclinical atrial fibrillation and the risk of stroke. N Engl J Med.

[51] Oral H, Veerareddy S, Good E (2004). Prevalence of asymptomatic recurrences of atrial fibrillation after successful radiofrequency catheter ablation. J Cardiovasc Electrophysiol.

[52] Vasamreddy CR, Dalal D, Dong J (2006). Symptomatic and asymptomatic atrial fibrillation in patients undergoing radiofrequency catheter ablation. J Cardiovasc Electrophysiol.

[53] Klemm HU, Ventura R, Rostock T (2006). Correlation of symptoms to ECG diagnosis following atrial fibrillation ablation. J Cardiovasc Electrophysiol.

[54] Hindricks G, Piorkowski C, Tanner H (2005). Perception of atrial fibrillation before and after radiofrequency catheter ablation: relevance of asymptomatic arrhythmia recurrence. Circulation.

[55] Saririan M, Eisenberg MJ (2003). Myocardial laser revascularization for the treatment of end-stage coronary artery disease. J Am Coll Cardiol.

[56] Wazni OM, Marrouche NF, Martin DO (2005). Radiofrequency ablation vs antiarrhythmic drugs as first-line treatment of symptomatic atrial fibrillation: a randomized trial. JAMA.

[57] Pappone C, Augello G, Sala S (2006). A randomized trial of circumferential pulmonary vein ablation versus antiarrhythmic drug therapy in paroxysmal atrial fibrillation: the APAF Study. J Am Coll Cardiol.

[58] Jaïs P, Cauchemez B, Macle L (2008). Catheter ablation versus antiarrhythmic drugs for atrial fibrillation: the A4 study. Circulation.

[59] Stabile G, Bertaglia E, Senatore G (2003). Feasibility of pulmonary vein ostia radiofrequency ablation in patients with atrial fibrillation: a multicenter study (CACAF pilot study). Pacing Clin Electrophysiol.

[60] Kottkamp H, Tanner H, Kobza R (2004). Time courses and quantitative analysis of atrial fibrillation episode number and duration after circular plus linear left atrial lesions: trigger elimina-tion or substrate modification: early or delayed cure?. J Am Coll Cardiol.

[61] Dagres N, Kottkamp H, Piorkowski C (2010). Influence of the dura-tion of Holter monitoring on the detection of arrhythmia recurrences after catheter ablation of atrial fibrillation: implications for patient follow-up. Int J Cardiol.

[62] Senatore G, Stabile G, Bertaglia E (2005). Role of transtelephonic electrocardiographic monitoring in detecting short-term arrhythmia recurrences after radiofrequency ablation in patients with atrial fibrillation. J Am Coll Cardiol.

[63] Piorkowski C, Kottkamp H, Tanner H (2005). Value of different follow-up strategies to assess the efficacy of circumferential pulmonary vein ablation for the curative treatment of atrial fibrilla-tion. J Cardiovasc Electrophysiol.

[64] Weerasooriya R, Khairy P, Litalien J (2011). Catheter ablation for atrial fibrillation: are results maintained at 5 years of follow-up?. J Am Coll Cardiol.

[65] Sorgente A, Tung P, Wylie J, Josephson ME (2012). Six year follow-up after catheter ablation of atrial fibrillation: a palliation more than a true cure. Am J Cardiol.

[66] Martinek M, Aichinger J, Nesser HJ, Ziegler PD, Purerfellner H (2007). New insights into long-term follow-up of atrial fibrillation ablation: full disclosure by an implantable pacemaker device. J Cardiovasc Electrophysiol.

[67] Bertaglia E, Tondo C, De Simone A (2010). Does catheter ablation cure atrial fibrillation? Single-procedure outcome of drug-refractory atrial fibrillation ablation: a 6-year multicentre experience.. Europace.

[68] Ouyang F, Tilz R, Chun J (2010). Long-term results of catheter ablation in paroxysmal atrial fibrillation: lessons from a 5-year follow-up. Circulation.

[69] Tzou WS, Marchlinski FE, Zado ES (2010). Long-term outcome after successful catheter ablation of atrial fibrillation. Circ Arrhythm Electrophysiol.

[70] Seidl K, Meisel E, Van Agt E (1998). Is the atrial high rate episode diagnostic feature reliable in detecting paroxysmal episodes of atrial tachyarrhythmias?. Pacing Clin Electrophysiol.

[71] Moya A, Sutton R, Ammirati F (2009). Guidelines for the diagnosis and management of syncope (version 2009). Eur Heart J.

[72] Hindricks G, Pokushalov E, Urban L (2010). Performance of a new leadless implantable cardiac monitor in detecting and quantifying atrial fibrillation: Results of the XPECT trial. Circ Arrhythm Electrophysiol.

[73] Hanke T, Charitos EI, Stierle U (2009). Twenty-four-hour holter monitor follow-up does not provide accurate heart rhythm status after surgical atrial fibrillation ablation therapy: up to 12 months experience with a novel permanently implantable heart rhythm monitor device. Circulation.

[74] Eitel C, Husser D, Hindricks G (2011). Performance of an implantable automatic atrial fibrillation detection device: impact of software adjustments and relevance of manual episode analysis. Europace.

[75] Pokushalov E, Romanov A, Corbucci G (2011). Use of an implantable monitor to detect arrhythmia recurrences and select patients for early repeat catheter ablation for atrial fibrillation: a pilot study. Circ Arrhythm Electrophysiol.

[76] Arya A, Piorkowski C, Sommer P, Kottkamp H, Hindricks G (2007). Clinical implications of various follow up strategies after catheter ablation of atrial fibrillation. Pacing Clin Electrophysiol.

